# Describing settings of care in the last 100 days of life for cancer decedents: a population‐based descriptive study

**DOI:** 10.1002/cam4.5291

**Published:** 2022-10-24

**Authors:** Abe Hafid, Michelle Howard, Colleen Webber, Ana Gayowsky, Mary Scott, Aaron Jones, Amy T. Hsu, Peter Tanuseputro, James Downar, Katrin Conen, Doug Manuel, Sarina R. Isenberg

**Affiliations:** ^1^ Department of Family Medicine McMaster University Hamilton Canada; ^2^ Ottawa Hospital Research Institute Ottawa Canada; ^3^ Bruyère Research Institute Ottawa Canada; ^4^ ICES uOttawa Ottawa Canada; ^5^ ICES McMaster University Hamilton Canada; ^6^ Health Research Methods Evidence and Impact, McMaster University Hamilton Canada; ^7^ Department of Medicine University of Ottawa Ottawa Canada; ^8^ Division of Palliative Care, Department of Medicine University of Ottawa Ottawa Canada; ^9^ Department of Medicine McMaster University Hamilton Canada; ^10^ Department of Family Medicine Ottawa Hospital Research Institute, University of Ottawa Ottawa Canada

**Keywords:** cancer, end‐of‐life, health administrative data, retrospective studies, terminal care

## Abstract

**Background:**

Few studies have described the settings cancer decedents spend their end‐of‐life stage, with none considering homecare specifically. We describe the different settings of care experienced in the last 100 days of life by individuals with cancer and how settings of care change as they approached death.

**Methods:**

A retrospective cohort study from January 2013 to December 2017, of decedents whose primary cause of death was cancer, using linked population‐level health administrative datasets in Ontario, Canada.

**Results:**

Decedents 125,755 were included in our cohort. The average age at death was 73, 46% were female, and 14% resided in rural regions. And 24% died of lung cancer, 7% breast, 7% colorectal, 7% pancreatic, 5% prostate, and 50% other cancers. In the last 100 days of life, decedents spent 25.9 days in institutions, 25.8 days receiving care in the community, and 48.3 days at home without any care. Individuals who died of lung and pancreatic cancers spent the most days at home without any care (52.1 and 52.6 days), while individuals who died of prostate and breast cancer spent the least days at home without any care (41.6 and 45.1 days). Regardless of cancer type, decedents spent fewer days at home and more days in institutions as they approached death, despite established patient preferences for an end‐of‐life experience at home.

**Conclusions:**

In the last 100 days of life, cancer decedents spent most of their time in either institutions or at home without any care. Improving homecare services during the end‐of‐life may provide people dying of cancer with a preferred dying experience.

## Graphical abstract

A population‐based descriptive study using linked health administrative data in Ontario, Canada, described where patients dying of cancer spent their last 100 days of life. From 2013 to 2017, patients dying with cancer spent most of their time in either institutions (25.9 days) or at home without any care (48.3 days).

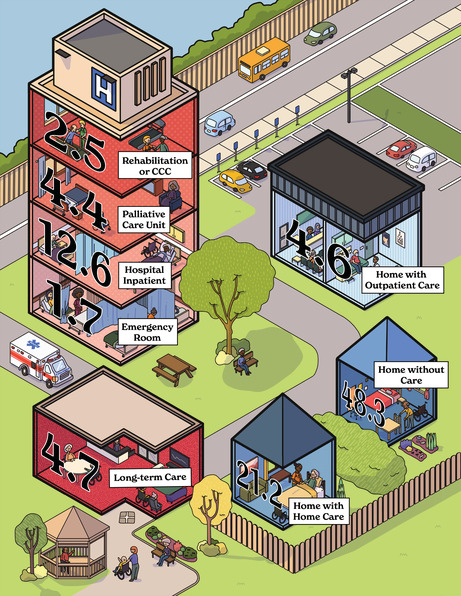

## 1 BACKGROUND

Patients with cancer have high care needs toward the end of life. Patients dying with cancer tend to have multiple comorbidities,^1^ and are more likely to experience severe pain at the end‐of‐life stage compared to other end‐of‐life trajectories.^2^ They also experience aggressive interventions during the end‐of‐life phase,^3^, ^4^ with almost a quarter of decedents who died of cancer in Ontario, Canada experiencing at least one aggressive intervention (i.e., receiving chemotherapy in the last 14 days of life, having more than one emergency room visit or hospitalization in the last 30 days of life).^3^ A retrospective cohort study of cancer decedents from 7 countries reported that between 44% to 60% of decedents experienced at least one hospitalization during the last 30 days of life and 5% to 13% of decedents received chemotherapy during this period as well.^5^

Research has been conducted on preferred settings of death for individuals with cancer. A qualitative study of patients dying with cancer reported that patients desire a comfortable end‐of‐life experience with limited suffering, maintaining their independence and autonomy, and, ultimately, dying in their own homes.^6^ A survey of bereaved caregivers in Ontario also reported that patients with terminal illnesses (e.g., cancer) overwhelmingly preferred to die at home as well.^7^ Similarly, a systematic review of 210 studies from over 33 countries identified that most individuals prefer to die at home instead of an institution and that most individuals' preferences remained static as they approached death.^8^ Despite this, 53% of cancer decedents die in a hospital setting in Ontario,^1^ thereby contradicting the established benefits of homecare during this phase^9^ and patient preferences in spending their final days at home.

Palliative care in Ontario—as provided by physicians and nurse practitioners‐‐ is delivered in the hospital (both in a consultative model and a dedicated palliative care unit), in complex continuing care/sub‐acute care (in the form of a palliative care unit), at outpatient clinics, in nursing homes, and in the home. Almost half of decedents receive at least 1 palliative care service in the last year of life, with the majority of it being delivered in acute care and outpatient settings.^10^ Hospice in Ontario refers only to dedicated facilities and there are only approximately 271 hospice beds in the entire province.^11^ Unfortunately, health services information for hospice facilities is not captured through health administrative datasets in Ontario, Canada. Nevertheless, a 2014 cross‐country comparison study estimated that 16% to 30% of decedents received hospice palliative care services in Canada, compared to 23% in England, 12% in Germany, and 41% in the United States.^12^ Few studies have explored which settings decedents spend their end‐of‐life stage in.^13^, ^14^, ^15^, ^16^ Of these studies, only two limited their study cohorts to cancer decedents, of which one was specific to hematologic cancers.^13^, ^15^ Cheung et al. and Andersen et al. both reported that cancer decedents spend the majority of their final days at home. However, they only studied the number of days spent at home in the last 6 months of life; neglecting to differentiate between days at home with home care and days at home without any care, nor reporting on days spent in other settings.

To our knowledge, no studies have described the different settings of care experienced by cancer decedents during the last 100 days of life using population‐level health administrative data. Therefore, the objective of this study was to describe the different settings of care experienced in the last 100 days of life by decedents whose primary cause of death was cancer and how settings of care change as the decedent approached death. The last 100 days of life is a timeframe of interest as healthcare utilization and cost increase significantly during this timeframe for decedents with a history of cancer.^17^ In addition, a systematic review of retrospective studies using linked‐health administrative datasets demonstrated that the use of various healthcare resources increased toward the end‐of‐life phase for patients dying with cancer.^5^, ^18^, ^19^

## 2 METHODS

### 2.1 Study design and data sources

We conducted a retrospective cohort study using linked population‐level health administrative datasets in Ontario, Canada, held at ICES (formerly known as the Institute for Clinical Evaluative Sciences). ICES is an independent, non‐profit research institute whose legal status under Ontario's health information privacy law allows it to collect and analyze health care and demographic data, without consent, for health system evaluation and improvement. Data holdings at ICES are derived from across all healthcare sectors in Ontario, with access to individual‐level data for services across the continuum of care. Datasets are linked using unique encoded identifiers and are analyzed internally at ICES (please see Supplementary File S1 for a description of datasets accessed). The use of data in this project was authorized under section 45 of Ontario's Personal Health Information Protection Act, which does not require review by a Research Ethics Board.

### 2.2 Population

The cohort initially consisted of all decedents aged 19 years or older at death who died between January 1, 2013, and December 31, 2017. Decedents were excluded if they were older than 105 years at death (in case of administrative error) if they were ineligible to the Ontario Health Insurance Plan (OHIP) during their last year of life,* if they had no healthcare encounters in the five years before death, and if they had an address outside Ontario at death. Additional data quality exclusions include decedents whose primary cause of death was not cancer or female decedents whose cause of death was prostate cancer.

Decedents were classified into six categories of major cancer sites for their cause of death: lung, breast, colorectal, pancreatic, prostate, and other. Cause of death was first captured through the Ontario Registrar General – Deaths database. Cancer site was identified via death data from the Ontario Cancer Registry. Please see Supplementary File S2 for the ICD10 diagnosis codes used to categorize cancer sites.

### 2.3 Measures

#### 2.3.1 Cohort characteristics

Decedent characteristics were captured at 100 days before death using information from the administrative databases. Age and sex were obtained from the Registered Persons Database (RPDB). Rurality status and neighborhood income level were collected by linking the decedents' postal code in RPDB to the 2011 Canadian census data if their death date occurred between 2012 and 2015, and 2016 Canadian census data if their death date occurred after 2016. Comorbidity status for conditions other than cancer was obtained using previously developed algorithms that use diagnosis codes and medication data to identify prevalent chronic conditions^20^, ^21^, ^22^, ^23^, ^24^, ^25^, ^26^, ^27^, ^28^, ^29^ and was captured using a 265‐day period, from the start of the last year of life to the first day of the last 100 days of life.

#### 2.3.2 Defining healthcare settings

Past studies have defined the end‐of‐life stage as the last 30 days of life^2^, ^3^, ^4^ or the last 180 days of life^1^, ^13^, ^14^, ^15^, ^16^; in this study, we define this stage as the last 100 days of life as healthcare utilization increases with an increasing focus on palliative care delivery. Therefore, this study described the settings in which decedents received care in their last 100 days of life, including days spent both in institutions and in the community. Days in institutions were categorized as emergency room, inpatient hospital, palliative care units, complex continuing care or rehabilitation, and long‐term care.

Days in the emergency room were derived from any encounter documented in the National Ambulatory Care Reporting System (NACRS) database, which included scheduled visits to and transfers from the ER, visits that resulted in an inpatient admission, and registrations that did not result in a physician encounter.

Days in inpatient hospital settings were derived from any encounter in the Discharge Abstract Database (DAD) and the Ontario Mental Health Reporting System metadata.

Palliative care units are exclusively located in acute care hospitals and complex continuing care facilities and differ from hospice facilities, which through dedicated facilities, are considered to be in the community. We used an approach to approximate whether an individual was in a palliative care unit. Days in palliative care units in complex continuing care facilities were captured using OHIP physician billings and the Continuing Care (CCRS). Specifically, an individual was considered to be admitted to a palliative care unit if at least 50% of the OHIP billing claims between a given admission and discharge date in CCRS were palliative in nature and if the individual had at least 1 encounter with a palliative care specialist. Palliative care specialists were identified using a validated algorithm that identified physicians as palliative care specialists if they billed 10% or more palliative fee codes during a 2‐year time frame.^30^ Days in palliative care units in acute hospitals were captured using DAD. An individual was considered to be admitted to a palliative care unit if the main patient service was palliative care and the most responsible diagnosis on the hospitalization record was palliative care (ICD‐10 diagnosis code Z51.5 for palliative care). This approach has been used in previous Ontario‐based studies.^31^, ^32^, ^33^

Days in complex continuing care or rehabilitation were derived from admission and discharge dates in CCRS and the National Rehabilitation Reporting System metadata respectively, while subtracting time spent in other settings if hospitalizations and ER visits fell within those dates. Days in long‐term care were also derived from admission and discharge dates from CCRS‐LTC, while subtracting time spent in other settings if hospitalizations and ER visits fell within those dates.

Days in the community were categorized as home with outpatient care, home with publicly funded home care (including physician home visits), and home without healthcare. Days at home with outpatient care were derived from OHIP billing fee codes whose location of service was office or phone. Days at home with home care were derived from OHIP billing fee codes whose location of service was home or from service dates from the Home Care Dataset. Consequently, days at home without any care were derived from the remaining days not spent in any of the above settings.

Please see Supplementary File S3 for a hierarchy order and administrative codes used to define settings of care.

### 2.4 Statistical analyses

Descriptive results are presented as counts and proportions for categorical variables, and as mean and standard deviations (SD) for continuous variables. Absolute and relative differences are presented for changes in days spent in different healthcare settings across the last 100 days of life. Results are reported by cancer type and socio‐demographic factors. All analyses were conducted using SAS Enterprise Guide v.7.15.

## 3 RESULTS

**FIGURE 1:**
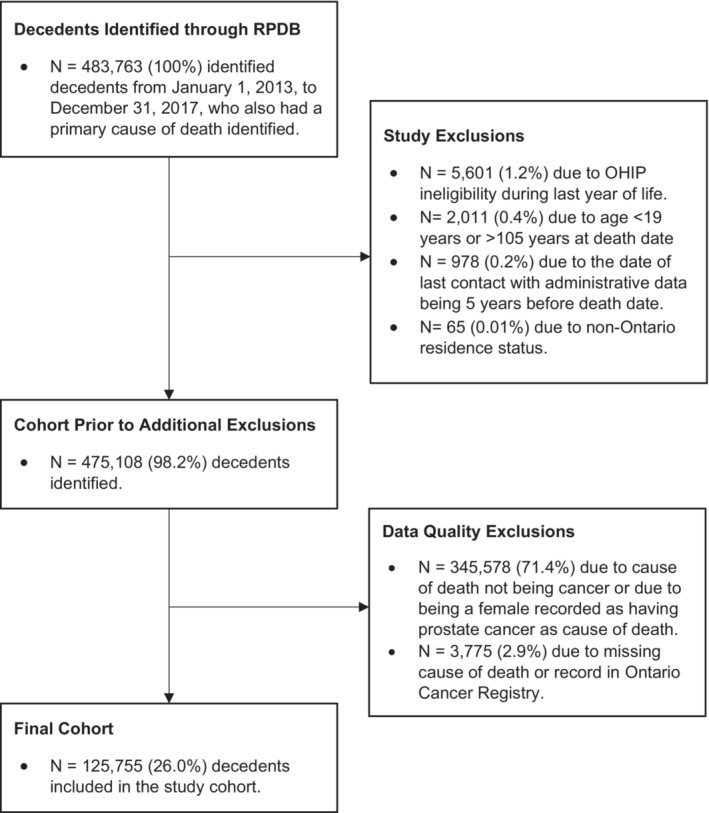
Cohort creation flow diagram

From January 1, 2013, to December 31, 2017, there were 475,108 decedents aged 19 years and older after 8655 excluding decedents ineligible for OHIP during the last year of life, decedents aged <19 years or > 105 years at death, decedents who did not have healthcare contact within the last 5 years of life, and decedents with non‐Ontario residence status. After excluding non‐cancer decedents, females with prostate cancer as their cause of death and missing cause of death information or record in the Ontario Cancer Registry, 125,755 decedents remained in the study cohort (Figure 1). The number of excluded females being recorded as having prostate cancer was suppressed in Figure 1 to prevent reidentification due to small cell issues. The mean age of decedents was 73.0 (SD 12.9), 46.1% were female, 14.1% resided in rural regions, and 86.2% had at least one other prevalent condition in addition to cancer before the last 100 days of life. Of the total cohort, 23.6% died of lung cancer, 7.2% breast cancer, 7.2% colorectal cancer, 6.6% pancreatic cancer, 5.2% prostate cancer, and 50.3% other cancer types, consisting of various cancers with small proportions preventing functional grouping (Table 1). Decedents experienced 1.7 emergency department visits and 1.2 inpatient hospital admissions in the last 100 days of life respectively.

**TABLE 1:** Profile of cancer decedents aged 19 years or older who died between January 1, 2013, and December 31, 2017, in Ontario, Canada

Variable	Cancer Type						Total Cohort (*n* = 125,755)
	Lung (*n* = 29,631)	Breast (*n* = 9021)	Colorectal (*n* = 9001)	Pancreatic (*n* = 8331)	Prostate (*n* = 6494)	Other (*n* = 63,277)	
Age in years, *n* (%)							
19–44	185 (0.6%)	480 (5.3%)	181 (2.0%)	92 (1.1%)	0 (0.0%)	1955 (3.1%)	2893 (2.3%)
45–54	1338 (4.5%)	1127 (12.5%)	550 (6.1%)	473 (5.7%)	78 (1.2%)	4308 (6.8%)	7874 (6.3%)
55–64	5388 (18.2%)	1754 (19.4%)	1230 (13.7%)	1464 (17.6%)	450 (6.9%)	10,455 (16.5%)	20,741 (16.5%)
65–74	9343 (31.5%)	1993 (22.1%)	2046 (22.7%)	2397 (28.8%)	1328 (20.4%)	15,596 (24.6%)	32,703 (26.0%)
75–84	9114 (30.8%)	1857 (20.6%)	2665 (29.6%)	2434 (29.2%)	2332 (35.9%)	17,720 (28.0%)	36,122 (28.7%)
85–94	4000 (13.5%)	1554 (17.2%)	2071 (23.0%)	1347 (16.2%)	2129 (32.8%)	12,086 (19.1%)	23,187 (18.4%)
95+	263 (0.9%)	256 (2.8%)	258 (2.9%)	124 (1.5%)	177 (2.7%)	1157 (1.8%)	2235 (1.8%)
Mean (SD)	72.6 (10.9)	69.7 (15.2)	74.71 (13.1)	72.9 (11.9)	79.7 (9.8)	72.6 (13.6)	73.0 (12.9)
Female, *n* (%)	14,084 (47.5%)	8948 (99.2%)	4390 (48.8%)	4002 (48.0%)	0 (0.0%)	29,157 (46.1%)	60,581 (48.2%)
Rural residence, *n* (%)	4537 (15.3%)	1139 (12.6%)	1316 (14.6%)	1101 (13.2%)	1048 (16.1%)	8606 (13.6%)	17,747 (14.1%)
Neighborhood Income Quintile, *n* (%)							
1 (lowest)	7623 (25.7%)	1944 (21.5%)	1973 (21.9%)	1761 (21.1%)	1362 (21.0%)	14,023 (22.2%)	28,686 (22.8%)
2	6790 (22.9%)	1931 (21.4%)	1915 (21.3%)	1776 (21.3%)	1318 (20.3%)	13,449 (21.3%)	27,179 (21.6%)
3	5715 (19.3%)	1777 (19.7%)	1802 (20.0%)	1602 (19.2%)	1297 (20.0%)	12,496 (19.7%)	24,689 (19.6%)
4	4944 (16.7%)	1643 (18.2%)	1678 (18.6%)	1565 (18.8%)	1244 (19.2%)	11,485 (18.2%)	22,559 (17.9%)
5 (highest)	4467 (15.1%)	1689 (18.7%)	1603 (17.8%)	1614 (19.4%)	1253 (19.3%)	11,600 (18.3%)	22,226 (17.7%)
Prevalent conditions in the last 1200 days of life, *n* (%)							
CHF	4273 (14.4%)	1159 (12.8%)	1331 (14.8%)	914 (11.0%)	1244 (19.2%)	9277 (14.7%)	18,198 (14.5%)
COPD	10,413 (35.1%)	826 (9.2%)	1265 (14.1%)	1010 (12.1%)	976 (15.0%)	8690 (13.7%)	23,180 (18.4%)
Renal Disease	2149 (7.3%)	561 (6.2%)	837 (9.3%)	596 (7.2%)	1093 (16.8%)	7197 (11.4%)	12,433 (9.9%)
Number of prevalent conditions in the last 100 days of life, *n* (%)							
0	3704 (12.5%)	1862 (20.6%)	1330 (14.8%)	1060 (12.7%)	565 (8.7%)	8784 (13.9%)	17,305 (13.8%)
1	6467 (21.8%)	2359 (26.2%)	2236 (24.8%)	1944 (23.3%)	1491 (23.0%)	14,950 (23.6%)	29,447 (23.4%)
2	7228 (24.4%)	2094 (23.2%)	2265 (25.2%)	2239 (26.9%)	1571 (24.2%)	15,738 (24.9%)	31,135 (24.8%)
3	5426 (18.3%)	1358 (15.1%)	1531 (17.0%)	1517 (18.2%)	1335 (20.6%)	11,128 (17.6%)	22,295 (17.7%)
4	3500 (11.8%)	758 (8.4%)	881 (9.8%)	819 (9.8%)	758 (11.7%)	6704 (10.6%)	13,420 (10.7%)
5	1882 (6.4%)	360 (4.0%)	448 (5.0%)	433 (5.2%)	436 (6.7%)	3484 (5.5%)	7043 (5.6%)
6+	1424 (4.8%)	230 (2.5%)	310 (3.4%)	319 (3.8%)	338 (5.2%)	2489 (3.9%)	5110 (4.1%)
Number of emergency department visits in the last 100 days of life, Mean (SD)	1.8 (1.5)	1.4 (1.4)	1.5 (1.5)	1.8 (1.6)	1.6 (1.6)	1.8 (1.6)	1.7 (1.6)
Number of inpatient hospital admissions in the last 100 days of life, Mean (SD)	1.2 (1.0)	1.0 (0.9)	1.1 (1.0)	1.2 (1.0)	1.1 (1.0)	1.3 (1.1)	1.2 (1.0)

Abbreviation: SD, Standard deviation.

**FIGURE 2:**
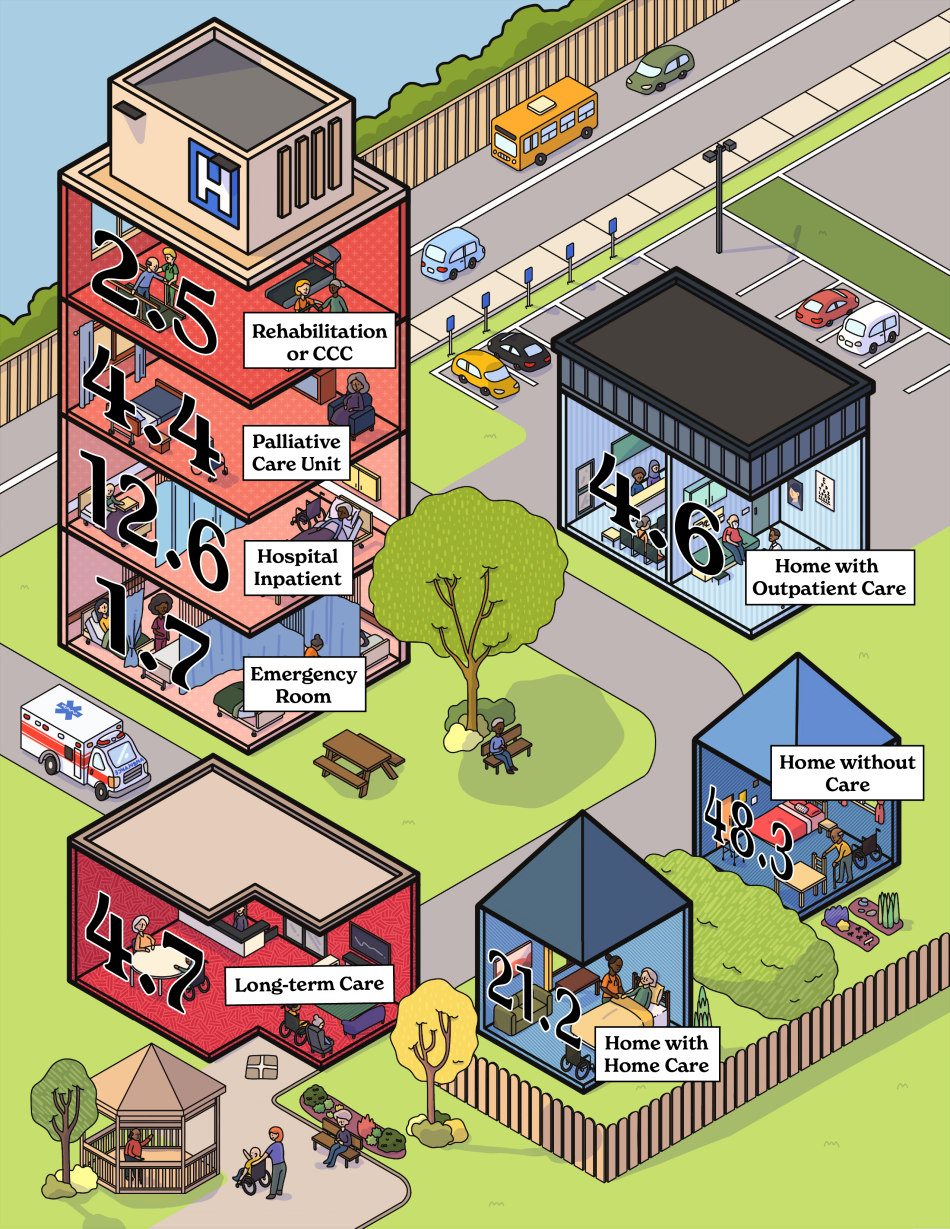
Mean number of days spent in healthcare settings in the last 100 days of life amongst cancer decedents (*n* = 125,755) in Ontario from 2013 to 2017. CCC, complex continuing care.

In the last 100 days of life, decedents spent a mean of 1.7 days in emergency rooms, 12.6 days in inpatient hospitals, 4.4 days in palliative care units, 2.5 days in complex continuing care or rehabilitation, 4.7 days in long‐term care, 4.6 days at home with outpatient care, 21.2 days at home with home care, and 48.3 days at home without any care (Figure 2). Of note, the median days were less than the means for all settings, except for days at home without healthcare (Supplementary File S4), suggesting a right skew to the data.

**FIGURE 3:**
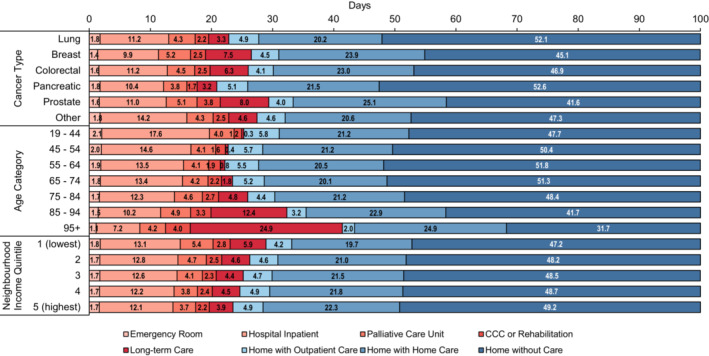
Mean number of days spent in healthcare settings in the last 100 days of life per cancer type, age category, and neighborhood income quintile, amongst cancer decedents (*n* = 125,755) in Ontario from 2013 to 2017. Note: Data labels report mean days spent in each respective settings in the last 100 days of life.

Results were stratified by cancer type, age category, and neighborhood income quintile (Figure 3). Lung cancer and pancreatic cancer decedents spent an average of 52.1 and 52.6 days at home without care, while prostate cancer and breast cancer decedents spent an average of 41.6 and 45.1 days at home without care. Decedents spent between an average of 3.8 (pancreatic) and 5.2 (breast) days in a palliative care unit, between 9.9 (breast) and 14.2 (other) days in an inpatient hospital, and between 3.2 (pancreatic) and 8.0 (prostate) days in long‐term care. As age increased, decedents spent fewer days at home without care (19–44 = 47.3 days; 95 + = 37.7 days) and in hospital inpatient settings (19–44 = 17.6 days; 95 + = 7.2 days). Days spent in long‐term care increased as decedents' age increased, with decedents aged 95+ spending 24.9 days in long‐term care. Differences in places of care according to neighborhood income quintile were marginal; however, there was a general pattern that as income increased, days in institutions decreased and days at home increased. Please see Supplementary File S5 for results stratified by both cancer type and age.

**FIGURE 4:**
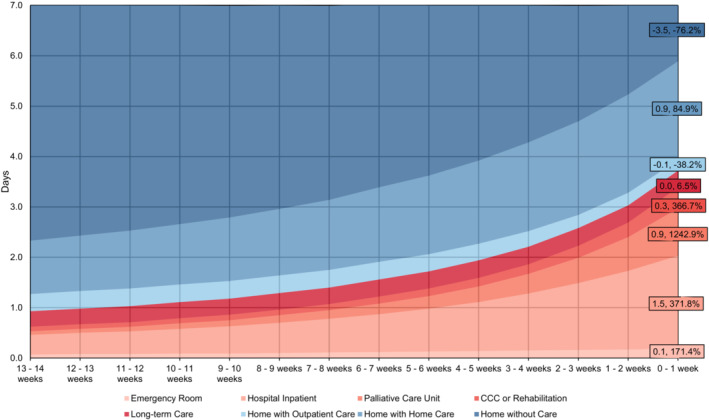
Mean Days spent in healthcare settings in the last 14 weeks of life (approximately last 100 days of life), amongst cancer decedents (*n* = 125,755) in Ontario from 2013 to 2017. Note: Data labels report absolute and relative difference in days from the last 14 week of life to the last week of life.

Places of care were analyzed weekly across the last 14 weeks of life (i.e., approximately the last 100 days) to measure changes in setting as decedents approached death (Figure 4). Absolute and relative differences were produced for each setting to measure change between 14 weeks away from death, until the last week before death. Overall, the average number of weekly days spent at home decreased while days in institutions increased as decedents approached death. Days at home without care decreased by 3.5 days, representing a 76% reduction, while days in hospital inpatient settings increased by 1.5 days, representing a 371% increase, and days at home with home care increased by 0.9 days, representing a 1242% increase. Please refer to Supplementary File S6 for results stratified by cancer type.

Transitions between home, hospital, long‐term care, and palliative care units, were captured from the last 4 months of life (Table 2). Between 4 months to 3 months before death, most decedents remained in their original location, while some moved from home to hospital (*n* = 6317; 5%) and hospital to home (*n* = 3040; 2%). Most decedents remained in their original setting as they approached death, while the percentage of decedents moving from home to hospital continued to increase in the last month (*n* = 22,612; 18%).

**TABLE 2:** Movement between different care settings in the last 4 months of life, for cancer decedents (*n* = 125,262) who died between January 1, 2013, and December 31, 2017, in Ontario, Canada

Source		Target	Months 4 to 3, *N* (%)	Months 3 to 2, *N* (%)	Months 2 to 1, *N* (%)	Total number of transitions
			*N* = 125,755	*N* = 125,755	*N* = 125,755	*N* = 377,265
Home	➔	Home	103,298 (82.1%)	92,504 (73.6%)	67,146 (53.4%)	262,948 (69.7%)
Home	➔	Hospital	6317 (5.0%)	11,655 (9.3%)	22,612 (18.0%)	40,584 (10.8%)
Home	➔	Long‐term care	188 (0.2%)	251 (0.2%)	222 (0.2%)	661 (0.2%)
Home	➔	Palliative care unit	753 (0.6%)	2095 (1.7%)	6410 (5.1%)	9258 (2.5%)
Hospital	➔	Home	3040 (2.4%)	3638 (2.9%)	4300 (3.4%)	10,978 (2.9%)
Hospital	➔	Hospital	4483 (3.6%)	6251 (5.0%)	11,645 (9.3%)	22,379 (5.9%)
Hospital	➔	Long‐term care	193 (0.6%)	255 (0.2%)	272 (0.2%)	720 (0.2%)
Hospital	➔	Palliative care unit	364 (0.3%)	754 (0.6%)	1865 (1.5%)	2983 (0.8%)
Long‐term care	➔	Home	27 (0.0%)	28 (0.0%)	36 (0.0%)	91 (0.0%)
Long‐term care	➔	Hospital	67 (0.1%)	119 (0.1%)	343 (0.3%)	529 (0.1%)
Long‐term care	➔	Long‐term care	5549 (4.4%)	5772 (4.6%)	5824 (4.6%)	17,145 (4.5%)
Long‐term care	➔	Palliative care unit	7 (0.0%)	18 (0.0%)	89 (0.1%)	114 (0.0%)
Palliative care unit	➔	Home	140 (0.1%)	220 (0.2%)	344 (0.3%)	704 (0.2%)
Palliative care unit	➔	Hospital	31 (0.0%)	57 (0.1%)	157 (0.1%)	245 (0.1%)
Palliative care unit	➔	Long‐term care	7 (0.0%)	14 (0.0%)	21 (0.0%)	42 (0.0%)
Palliative care unit	➔	Palliative care unit	1291 (1.0%)	2124 (1.7%)	4469 (3.6%)	7884 (2.1%)

*Note*: Decedents who did not change care settings have consistent source and target settings (i.e., home to home, hospital to hospital, long‐term care to long‐term care home, palliative care unit to palliative care unit).

## 4 DISCUSSION

In this retrospective study describing the different settings of care experienced in the last 100 days of life in Ontario, Canada, cancer decedents spent almost half of their final days at home without receiving any care, 25 days in institutions, and 21 days at home with home care services, including physician home services. Further, decedents increasingly spent more days in institutions as they approached death, resulting in fewer days spent at home consequently.

Cancer decedents of less aggressive cancers, like breast, colorectal, and prostate, spent more days in institutions compared to decedents with more aggressive cancers like pancreatic and lung cancers. These findings may reflect the differences in treatment on survival rates between less and more aggressive cancers. For instance, people with breast, colorectal, and prostate cancers, have significantly higher five‐ and 10‐year survival rates than people with lung or pancreatic cancers,^34^ demonstrating the differences in treatment effectiveness between cancers. In turn, people dying from less aggressive cancers may spend more time in institutions as they may receive more treatment. This may be further compounded by aggressive cancers, such as lung and pancreatic cancers, rapidly progressing which may make it difficult for physicians to coordinate homecare or palliative care. Nevertheless, further statistical analyses would be required to verify that differences between cancer types are not attributed to random variation. Regardless of cancer type, decedents spent more days in institutions as they approached death, complementing existing literature.^14^ Further, our findings demonstrate that almost one in five decedents transitioned from home to hospital in the last month of life, which reinforces previous findings as well.^35^, ^36^

Despite transitioning to hospital settings, decedents spent little time in palliative care units that provide end‐of‐life care to referred patients. This may be attributed to there only being 31 palliative care units in Ontario and few in rural areas.^37^ As a result, rural decedents may experience difficulties in accessing palliative care units, as described in the literature.^38^ Further, limited time spent in palliative care units may be due to the units having limited financial support and inadequate human resources,^37^ which may prevent higher uptake.

Our findings highlight the role of homecare services for people dying from cancer in Ontario, Canada. While there was an increase in days at home with home care toward death, most cancer decedents spent their final days either in an institution or at home without any care. Palliative home care resources are stretched, and many people lack the social supports to achieve a home death, even when resources are available. Increasing access to homecare services at the end‐of‐life can have positive outcomes, such as decreased acute care usage^39^, ^40^ and providing patients with a dignified end‐of‐life experience by maintaining patient autonomy and integrity,^41^ while also aligning with patient preferences and values regarding their preferred location of death.^4^, ^6^ Therefore, our findings, coupled with the literature, emphasize the importance of improving end‐of‐life care delivery to keep patients in the community and out of institutions during this important life stage.

### 4.1 Strengths and limitations

Since December 2017, the federal and provincial governments have developed frameworks for improving palliative care provision.^42^, ^43^ Along with these frameworks, the Ontario Palliative Care Teams were developed in 2017 to improve access to palliative home care, whose impacts may not be captured in our study as we used health administrative data dating from January 1, 2013, until December 31, 2017. Also, cause of death information was only available until 2017, as a result, we limited our decedent cohort to 2017 to enable us to categorize decedents into specific cancer sub‐groups. Therefore, our findings may not reflect the current standard of end‐of‐life care which may have resulted in cancer decedents receiving more homecare in their last 100 days of life. In addition, our study did not explore how our outcomes of interest varied by important decedent characteristics such as race, ethnicity, religious background, or gender. This information is unavailable at the individual‐level using health administrative datasets in Ontario. Moreover, this study was conducted using health administrative data from one Canadian province, which may not reflect the end‐of‐life experience of cancer decedents from other jurisdictions; however, we believe there are similarities to other regions with similar health systems. Further, this study uses a definition for identifying care in a palliative care unit that is not validated. It is often assumed that patients prefer to be at home; however, for various reasons, some patients may prefer to receive care at and die in an institution, and often only people with financial resources and strong social support networks have the privilege of dying at home. In addition, we do not have information on whether decedents had additional support in the home—either provided by private services or family or friends, which would impact whether decedents were able to stay at home. Regardless, this study adds granular information over time about the last 100 days of life for cancer decedents and their transitions across care settings.

## 5 CONCLUSION

In the last 100 days of life, people dying of cancer in Ontario, Canada, spend 25 days in institutions and nearly 50 days at home without any care, and only 21 days at home with home care services, including physician home visits. As individuals approach death, they increasingly spend more time in institutions instead of in the community, contradicting established patient preferences. It is unlikely or desirable to avoid all institutional admissions during the end‐of‐life. At a population level, increasing days spent in acute care indicate that we can expect a certain number of transitions to acute care, despite the receipt of homecare. This realization may prompt decision‐makers to shift resources to where needed most instead of a blanket approach in increasing services based on a diagnosis; for example, the often‐recommended bolstering of homecare or physician home visits to address the deterioration during the end‐of‐life stage.

## AUTHOR CONTRIBUTIONS

**Abe Hafid:** Conceptualization (equal); investigation (equal); methodology (equal); project administration (lead); visualization (lead); writing – original draft (lead); writing – review and editing (equal). **Michelle Howard:** Conceptualization (equal); funding acquisition (lead); investigation (equal); methodology (equal); writing – review and editing (equal). **Colleen Webber:** Conceptualization (equal); investigation (equal); methodology (equal); writing – review and editing (equal). **Ana Gayowsky:** Formal analysis (equal); methodology (equal); writing – review and editing (equal). **Mary Scott:** Methodology (equal); writing – review and editing (equal). **Aaron Jones:** Methodology (equal); writing – review and editing (equal). **Amy T Hsu:** Funding acquisition (equal); methodology (equal); writing – review and editing (equal). **Peter Tanuseputro:** Funding acquisition (equal); methodology (equal); writing – review and editing (equal). **James Downar:** Funding acquisition (equal); methodology (equal); writing – review and editing (equal). **Katrin Conen:** Methodology (equal); writing – review and editing (equal). **Doug Manuel:** Funding acquisition (equal); methodology (equal); writing – review and editing (equal). **Sarina R Isenberg:** Conceptualization (equal); funding acquisition (equal); investigation (equal); methodology (equal); writing – review and editing (equal).

## FUNDING INFORMATION

This study was funded by a grant from the Canadian Institutes of Health Research project #159771. This study was supported by ICES, which is funded by an annual grant from the Ontario Ministry of Health and Long‐Term Care. The *funders* had no *role* in study design, data collection and analysis, decision to publish, or preparation of the manuscript.

## ETHICS

The use of data in this project was authorized under section 45 of Ontario's Personal Health Information Protection Act, which does not require review by a Research Ethics Board.

## CONFLICT OF INTEREST

All authors declare that they have no competing interests.

## Supporting information


Appendix S1

Appendix S2

Appendix S3

Appendix S4

Appendix S5

Appendix S6

Appendix S7

## Data Availability

The data set from this study is held securely in coded form at ICES. While data sharing agreements prohibit ICES from making the data set publicly available, access may be granted to those who meet prespecified criteria for confidential access, available at www. ices.on.ca/DAS. The full data set creation plan and underlying analytic code are available from the authors upon request, understanding that the computer programs may rely upon coding templates or macros that are unique to ICES and are therefore either inaccessible or may require modification.

## ENDNOTE

* Almost all residents in Ontario are covered by the Ontario Health Insurance Plan, which covers physician services and hospital care. Eligible residents include citizens, permanent residents, landed immigrants and refugees, and indigenous Canadians. Very few people are not covered by the provincial health insurance plan.
