# Supplementary material for: Describing settings of care in the last 100 days of life for cancer decedents: a population‐based descriptive study
Source: Cancer Med. 2022 Oct 24;12(4):4809–20. doi: 10.1002/cam4.5291 (PMC9972173; doi:10.1002/cam4.5291)
Supplement: Supplementary file 2 — Appendix S2 [file CAM4-12-4809-s003.docx]

Supplementary File 1: Description of health administrative databases held at ICES used in study.

| **Database** | **Content** |
| --- | --- |
| ICES-derived cohorts | Validated cohorts of individuals with specific diseases and conditions. These include: Ontario Asthma Dataset (ASTHMA); Congestive Heart Failure (CHF) database; Chronic Obstructive Pulmonary Disease (COPD) database; Ontario Dementia Dataset (DEMENTIA); Ontario Hypertension Dataset (HYPER); Ontario Crohn’s and Colitis Cohort Dataset (OCCC); Ontario Diabetes Dataset (ODD); Ontario Myocardial Infarction Dataset (OMID); and the Ontario Rheumatoid Arthritis Dataset (ORAD). |
| Ontario Health Insurance Plan database (OHIP) | These data record all claims by Ontario physicians for inpatient and ambulatory visits, consultations and procedures. The data also include claims from optometrists for publicly-funded reimbursement and from laboratories for all diagnostic tests performed. |
| Ontario Registered Persons Database (RPDB) | Demographic, place of residence and vital status information for all persons eligible to receive insured health services in the province, including date of birth, sex, home address. |
| Ontario Registrar General – Deaths (ORGD) database | This database contains information (demographic, place of death, cause of death) for all decedents in Ontario. |
| Ontario Cancer Registry (OCR) | This database contains information (diagnoses, consultations/referrals, acute care use, cause of death) for all Ontario residents diagnosed with cancer and decedents who have died of cancer. |
| Home Care Database (HCD) | This dataset contains clinical information for home care recipients. Information includes assessments, program admission dates, and service records. |
| Ontario Drug Benefit Claims (ODB) | This database contains information (recipients, payments, claims, practitioners) for the Ontario Drug Benefit Program. |
| Discharge Abstract Database (DAD) | This data captures patient-level information (administrative, clinical, and demographic) on hospital discharges. Discharges include deaths, sign-outs, and transfers to other healthcare settings. |
| Client Agency Program Enrolment (CAPE) Dataset | This dataset details a list of patients registered to a primary care organization and identifies association with a specific primary care physician and what type of primary care organization. |
| Continuing Care Reporting System (CCRS) | This dataset contains information for all patients staying in a designated complex continuing care bed. These individuals are typically deemed to be in a non-acute state, but still in need for treatment (e.g., rehabilitation) in an institution |
| National Rehabilitation Reporting System (NRS) | This dataset contains information from participating adult inpatient rehabilitation facilities and programs across Ontario. |
| National Ambulatory Care Reporting System (NACRS) | This dataset captures all emergency department visits in Ontario. |
