# Supplementary material for: Describing settings of care in the last 100 days of life for cancer decedents: a population‐based descriptive study
Source: Cancer Med. 2022 Oct 24;12(4):4809–20. doi: 10.1002/cam4.5291 (PMC9972173; doi:10.1002/cam4.5291)
Supplement: Supplementary file 3 — Appendix S3 [file CAM4-12-4809-s004.docx]

| **Cancer Group** | **ICD10 Diagnosis Codes** |
| --- | --- |
| Lung | 1. C34.0 Malignant neoplasm of main bronchus. 2. C34.1 Malignant neoplasm of upper lobe, bronchus, or lung. 3. C34.2 Malignant neoplasm of middle lobe, bronchus, or lung. 4. C34.3 Malignant neoplasm of lower lobe, bronchus, or lung. 5. C34.8 Malignant neoplasm of overlapping sites of bronchus and lung. 6. C34.9 Malignant neoplasm of unspecified part of bronchus or lung. |
| Breast | 1. C50.0 Malignant neoplasm of nipple and areola. 2. C50.1 Malignant neoplasm of central portion of breast. 3. C50.2 Malignant neoplasm of upper-inner quadrant of breast. 4. C50.3 Malignant neoplasm of lower-inner quadrant of breast. 5. C50.4 Malignant neoplasm of upper-outer quadrant of breast. 6. C50.5 Malignant neoplasm of lower-outer quadrant of breast. 7. C50.6 Malignant neoplasm of axillary tail of breast. 8. C50.8 Malignant neoplasm of overlapping sites of breast. 9. C50.9 Malignant neoplasm of breast of unspecified site. |
| Colorectal | 1. C18.0 Malignant neoplasm of cecum. 2. C18.1 Malignant neoplasm of appendix. 3. C18.2 Malignant neoplasm of ascending colon. 4. C18.3 Malignant neoplasm of hepatic flexure. 5. C18.4 Malignant neoplasm of transverse colon. 6. C18.5 Malignant neoplasm of splenic flexure. 7. C18.6 Malignant neoplasm of descending colon. 8. C18.7 Malignant neoplasm of sigmoid colon. 9. C18.8 Malignant neoplasm of overlapping sites of colon. 10. C18.9 Malignant neoplasm of colon, unspecified. |
| Pancreatic | 1. C25.0 Malignant neoplasm of head of pancreas. 2. C25.1 Malignant neoplasm of body of pancreas. 3. C25.2 Malignant neoplasm of tail of pancreas. 4. C25.3 Malignant neoplasm of pancreatic duct. 5. C25.4 Malignant neoplasm of endocrine pancreas. 6. C25.7 Malignant neoplasm of other parts of pancreas. 7. C25.8 Malignant neoplasm of overlapping sites of pancreas. 8. C25.9 Malignant neoplasm of pancreas, unspecified. |
| Prostate | C61 Malignant neoplasm of prostate |
| Other | All remaining C00 to D48, excluding benign neoplasms (D10 to D36). |
| Note: All respective subcodes were included for the above listed ICD10 codes. | |

Supplementary File 2: ICD10 diagnosis codes used for cancer group categories
