# Supplementary material for: Describing settings of care in the last 100 days of life for cancer decedents: a population‐based descriptive study
Source: Cancer Med. 2022 Oct 24;12(4):4809–20. doi: 10.1002/cam4.5291 (PMC9972173; doi:10.1002/cam4.5291)
Supplement: Supplementary file 4 — Appendix S4 [file CAM4-12-4809-s002.docx]

Supplementary File 3: Describing hierarchy order for overlapping healthcare settings.

| **Order** | **Setting** | **Description** |
| --- | --- | --- |
| 1. | Emergency Room | If an emergency room visit was listed in National Ambulatory Care Reporting System, the day was listed as a day in an emergency room. |
| 2. | Palliative Care Unit | If a palliative care unit visit was listed, the day was listed as a day in a palliative care unit.  Palliative care unit visits were defined using the following Ontario Health Insurance Plan (OHIP) billing codes: A900, A901, A945, B960, B961, B962, B963, B964, B066, B986, B987, B988, B990, B992, B993, B994, B996, B997, B998, G511, G512, K015, K023, K007, C882, C945, C982, W872, W882, W972, and W982. |
| 3. | Inpatient Hospital | If an inpatient hospital visit was listed in the Discharge Abstract Database or the Ontario Mental Health Reporting System, the day was listed as a day in an inpatient hospital. |
| 4. | Complex Continuing Care, or Rehabilitation | If there was neither emergency room, palliative care unit, or inpatient hospital visits listed, the day was labelled as complex continuing care if there was a record in the Continuing Care Reporting System, and rehabilitation if there was a record in the National Rehabilitation Reporting System. |
| 5. | Long-term Care | If there was a long-term care record in the Continuing Care Reporting System, then the place of care was defined as long-term care.  However, if there was a stretch of days in complex continuing care, rehabilitation, or long-term care, followed by another stretch in either setting, overlapping days would be labelled as the following healthcare setting. Also, if there was a complex continuing care stay during a longer long-term care stay, the day would be labelled as complex continuing care. |
| 6. | Home with Outpatient Care | If there were no days listed in any institution but there were OHIP billing fee codes with locations ‘Office’ or ‘Phone’ or the OHIP billing code K700, the day was listed as a day at home with outpatient care. |
| 7. | Home with Home Care | If there were no days listed in any institution nor any billing fee codes for outpatient care but there was a Homecare Database record or an OHIP billing fee code with location ‘Home’, the day was listed as a day at home with home care. |
| 8. | Home without Healthcare | If there were none of the above listed records were documented, then the day was listed as a day at home without healthcare. |
