# Supplementary material for: Describing settings of care in the last 100 days of life for cancer decedents: a population‐based descriptive study
Source: Cancer Med. 2022 Oct 24;12(4):4809–20. doi: 10.1002/cam4.5291 (PMC9972173; doi:10.1002/cam4.5291)
Supplement: Supplementary file 5 — Appendix S5 [file CAM4-12-4809-s005.docx]

Supplementary File 4: Median number of days spent in healthcare settings in the last 100 days of life amongst cancer decedents (n= 125,755) in Ontario from 2013 to 2017.

| **Setting of care** | **Mean ± SD** | **Median (IQR)** |
| --- | --- | --- |
| Emergency Room | 1.7 (1.6) | 1 (1 - 2) |
| Hospital Inpatient | 12.6 (16.5) | 7 (0 - 18) |
| Palliative Care Unit | 4.4 (14.0) | 0 (0 - 0) |
| CCC or Rehabilitation | 2.5 (11.1) | 0 (0 - 0) |
| Long-term Care | 4.7 (20.3) | 0 (0 - 0) |
| Home with Outpatient Care | 4.6 (4.3) | 4 (1 - 7) |
| Home with Home Care | 21.2 (24.4) | 13 (1 - 32) |
| Home without Care | 48.3 (29.1) | 54 (25 - 72) |
