# Supplementary material for: Describing settings of care in the last 100 days of life for cancer decedents: a population‐based descriptive study
Source: Cancer Med. 2022 Oct 24;12(4):4809–20. doi: 10.1002/cam4.5291 (PMC9972173; doi:10.1002/cam4.5291)
Supplement: Supplementary file 6 — Appendix S6 [file CAM4-12-4809-s007.pdf]

Supplementary File 5: Mean number of days spent in healthcare settings in the last 100 days of life per cancer type and age, amongst cancer decedents (n=125,262) in Ontario from 2013 to 2017.

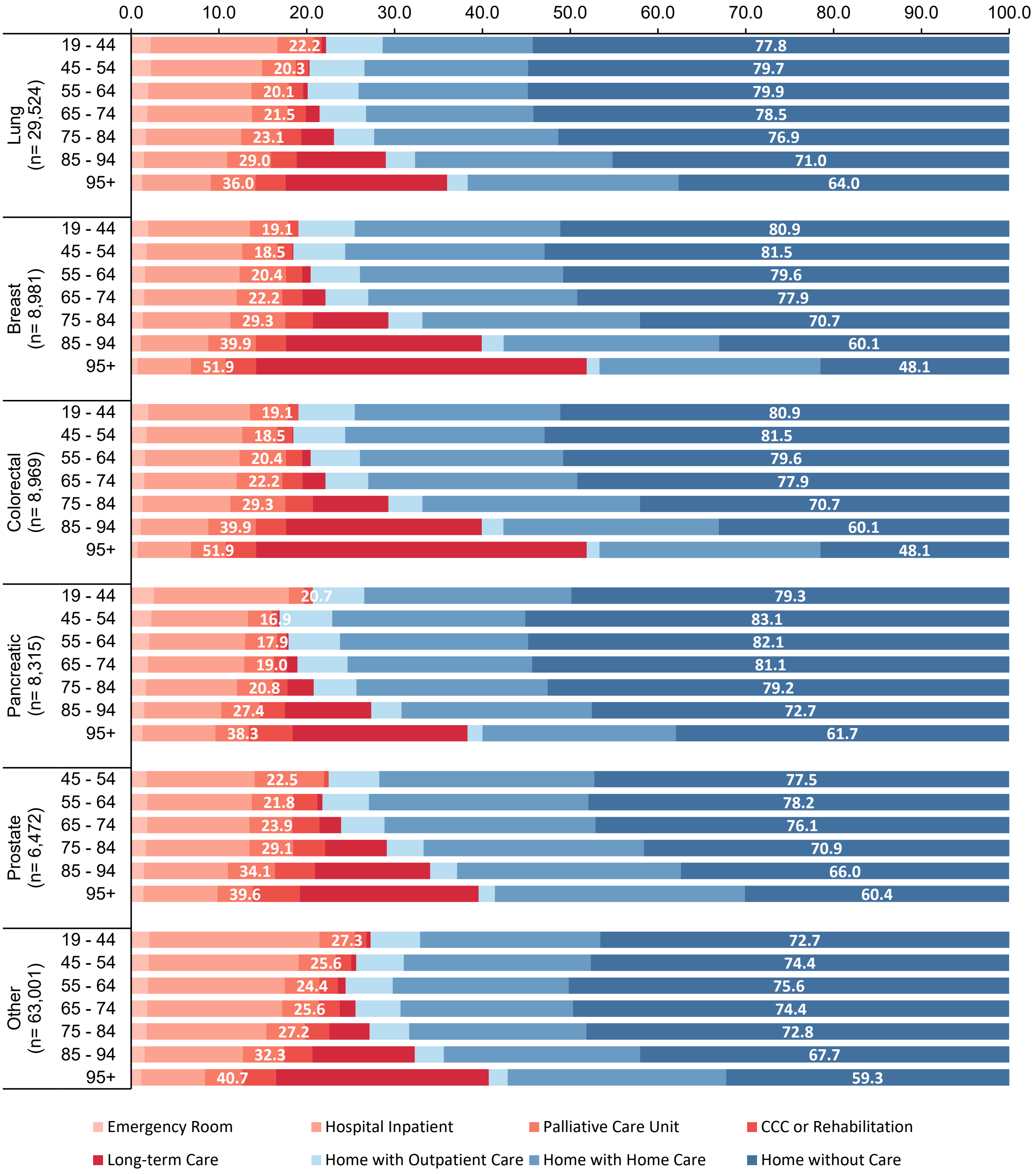

Note: Mean number of days in any institution and in the community are reported.
