# Supplementary material for: Describing settings of care in the last 100 days of life for cancer decedents: a population‐based descriptive study
Source: Cancer Med. 2022 Oct 24;12(4):4809–20. doi: 10.1002/cam4.5291 (PMC9972173; doi:10.1002/cam4.5291)
Supplement: Supplementary file 7 — Appendix S7 [file CAM4-12-4809-s001.pdf]

Supplementary File 6: Mean days spent in healthcare settings in the last 14 weeks of life stratified by cancer type, amongst cancer decedents (n= 125,755) in Ontario from 2013 to 2017.

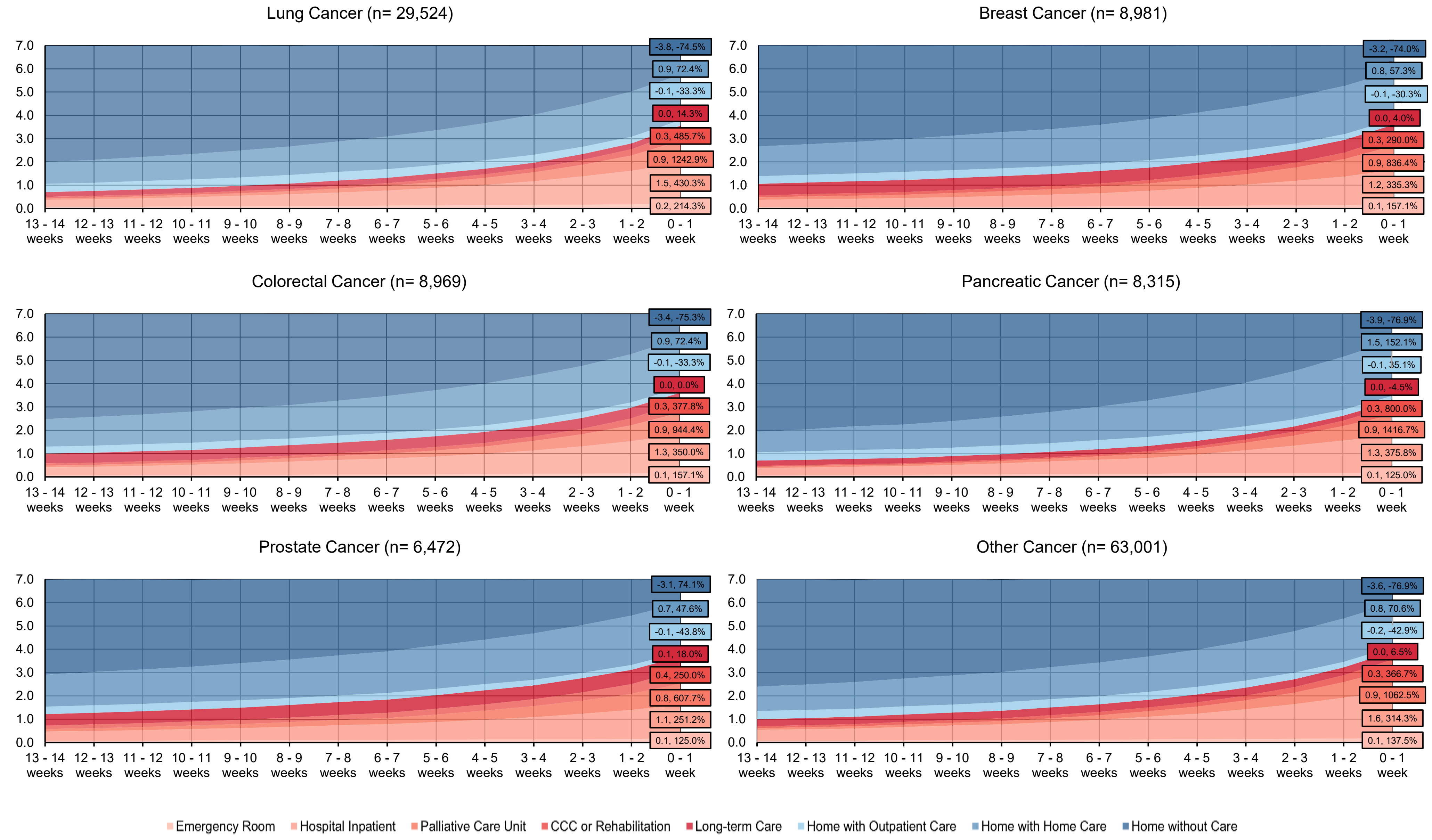

Note: Data labels report absolute and relative difference in days from the last 14 week of life to the last week of life.
